# The Emerging Role of T-Cell Immunoglobulin Mucin-3 in Breast Cancer: A Promising Target For Immunotherapy

**DOI:** 10.3389/fonc.2021.723238

**Published:** 2021-08-24

**Authors:** Yizi Cong, Jing Liu, Gang Chen, Guangdong Qiao

**Affiliations:** ^1^Department of Breast Surgery, The Affiliated Yantai Yuhuangding Hospital of Qingdao University, Yantai, China; ^2^Department of Pathology, The Affiliated Yantai Yuhuangding Hospital of Qingdao University, Yantai, China

**Keywords:** breast neoplasm, T-cell immunoglobulin mucin 3, prognosis, regulation, immunotherapy

## Abstract

Cancer treatment through immune checkpoint receptor blockade has made significant advances in the recent years. However, resistance to the current immune checkpoint inhibitors (ICIs) has been observed in many patients, who consequently do not respond to these treatments. T-cell immunoglobulin mucin-3 (Tim-3) is a novel immune checkpoint molecule emerging as a potential therapeutic target for cancer immunotherapy. Epidemiologic findings reveal that genetic polymorphisms in the Tim-3 gene are associated with increased susceptibility to breast cancer. In patients with breast cancer, Tim-3 is expressed both on immune and tumor cells. Accumulating evidence demonstrates that Tim-3 can notably affect breast cancer treatment outcome and prognosis. Therefore, Tim-3 is being regarded as a high-potential target for improving breast cancer therapy. In this review, we summarize the role of Tim-3 in breast cancer and the regulation mechanisms of Tim-3 to furnish evidences for future research and therapy.

## Introduction

Breast cancer is the most common malignant tumor and the leading cause of cancer-associated mortality among women (1). Although comprehensive therapies exist, patient response to the treatments significantly varies, which partly attributed to varying antitumor immune responses (2). Immunotherapy is being recognized as a key therapeutic modality for cancer and represents one of the most promising therapies. An increasing body of evidence suggests immune checkpoint molecules, such as programmed cell death protein 1 (PD-1), cytotoxic T-lymphocyte-associated protein 4 (CTLA-4), T cell immunoglobulin-3 (Tim-3, also known as Hepatitis A virus cellular receptor 2 [HAVCR2]), and lymphocyte activation gene-3 (LAG-3) are crucial regulators of immune escape and have critical roles in maintaining immune stability. This supports the development of immune checkpoint-targeting based therapeutic strategies (3). Following the success of immune checkpoint inhibitors (ICIs) in melanoma in 2010, multiple monoclonal antibodies against CTLA-4, PD-1, and programmed cell death 1 ligand 1(PD-L1) have been trialed and approved in solid tumors (4). Patients with metastatic breast cancer have shown an objective response rate of 21.4-39.4% receiving treatment with ICIs in clinical trials (5–7), indicating immunotherapy against the ICIs is a promising efficiency in metastatic breast cancer. However, many patients are still resistance to these targeted therapies (8). The second interim analysis of IMpassion130 indicates no significant difference in overall survival (OS) between atezolizumab plus nab-paclitaxel group and placebo plus nab-paclitaxel group in locally advanced or metastatic triple-negative breast cancer (TNBC), although it suggests a clinically OS benefit in patients with PD-L1 immune cell-positive disease (9). Therefore, intensive research on other inhibitory receptors is being conducted. Recent findings show that Tim-3 is part of a module that contains multiple coinhibitory receptors, which are coexpressed and coregulated on dysfunctional or “exhausted” T cells in cancer (10). A study showed that resistance to anti-CTLA-4 or anti PD-1/PD-L1 inhibitors is compensated by upregulation of other immune checkpoints, such as Tim-3 (11). Consequently, Tim-3 has gained prominence as a potential candidate for cancer immunotherapy. Blocking Tim-3 with other checkpoint inhibitors has been shown to enhance antitumor immunity and suppress tumor growth in several preclinical tumor models (12). These promising results indicate that Tim-3 could be a target for tumor therapy.

As a type I transmembrane protein, Tim-3 was discovered during attempts to identify new cell surface molecules for Th1 and Tc1 cells that produce IFN-γ (13). The Tim-3 locus, along with Tim-1 and Tim-4 loci, is located at 11B1.1 in the mouse genome and at 5q33.2 in the human genome (14). All Tim family molecules, except Tim-4, include a C-terminal cytoplasmic tail with a conserved tyrosine-based signaling motif. Unlike other checkpoint receptors such as PD-1 and T-cell immunoreceptor with Ig and ITIM domains (TIGIT), Tim-3 does not contain the classic inhibitory immunoreceptor tyrosine-based inhibition or immunoreceptor tyrosine-based switch signaling motifs in its cytoplasmic tail (15). Tim-3 inhibits cell proliferation, attenuates effective cytokine synthesis, and promotes apoptosis of activated T cells, by interacting with its ligands that bind to the Tim-3 extracellular immunoglobulin V domain (16). Four distinct ligands for Tim-3 have been identified currently: galectin-9 (Gal-9), phosphatidylserine (PtdSer), high-mobility group protein B1 (HMGB1), and CEACAM-1. A previous review has described the various interaction mechanisms between Tim-3 and its ligands (10).

Tim-3 is significantly upregulated in breast tumor tissues than in the normal tissues (17, 18), and is extremely highly expressed in basal-like and HER2-enriched breast cancer (19). Therefore, targeting Tim-3 has received much attention, particularly in TNBC. Tim-3 is not only expressed on IFN-γ-producing T cells, FoxP3^+^ Treg cells, macrophages, and dendritic cells (12), but also overexpressed on breast tumor cells (20, 21), which is associated with poor prognosis in breast cancer (20). The current review will focus on the emerging roles of Tim-3 in breast cancer and its regulating mechanisms with the aim to inform future research and therapeutic strategies.

## Genetic Polymorphisms in Tim-3 Increase Susceptibility to Breast Cancer

Single nucleotide polymorphisms (SNPs) represent a very common genetic variation in the human genome (22). SNPs in genes regulating DNA mismatch repair, cell cycle regulation, metabolism, and immunity are associated with genetic predisposition to cancer (22). Previous findings have shown that multiple polymorphisms in the promoter region (−574G/T, −882C/T, −1516G/T, and −1541C/T) and in the coding region (+4259T/G, amino acid substitution: Arg to Leu) of the Tim-3 gene were associated with several types of malignant tumors such as non-small-cell lung cancer (23), pancreatic cancer (24), and gastric cancer (25).

**Figure 1:**
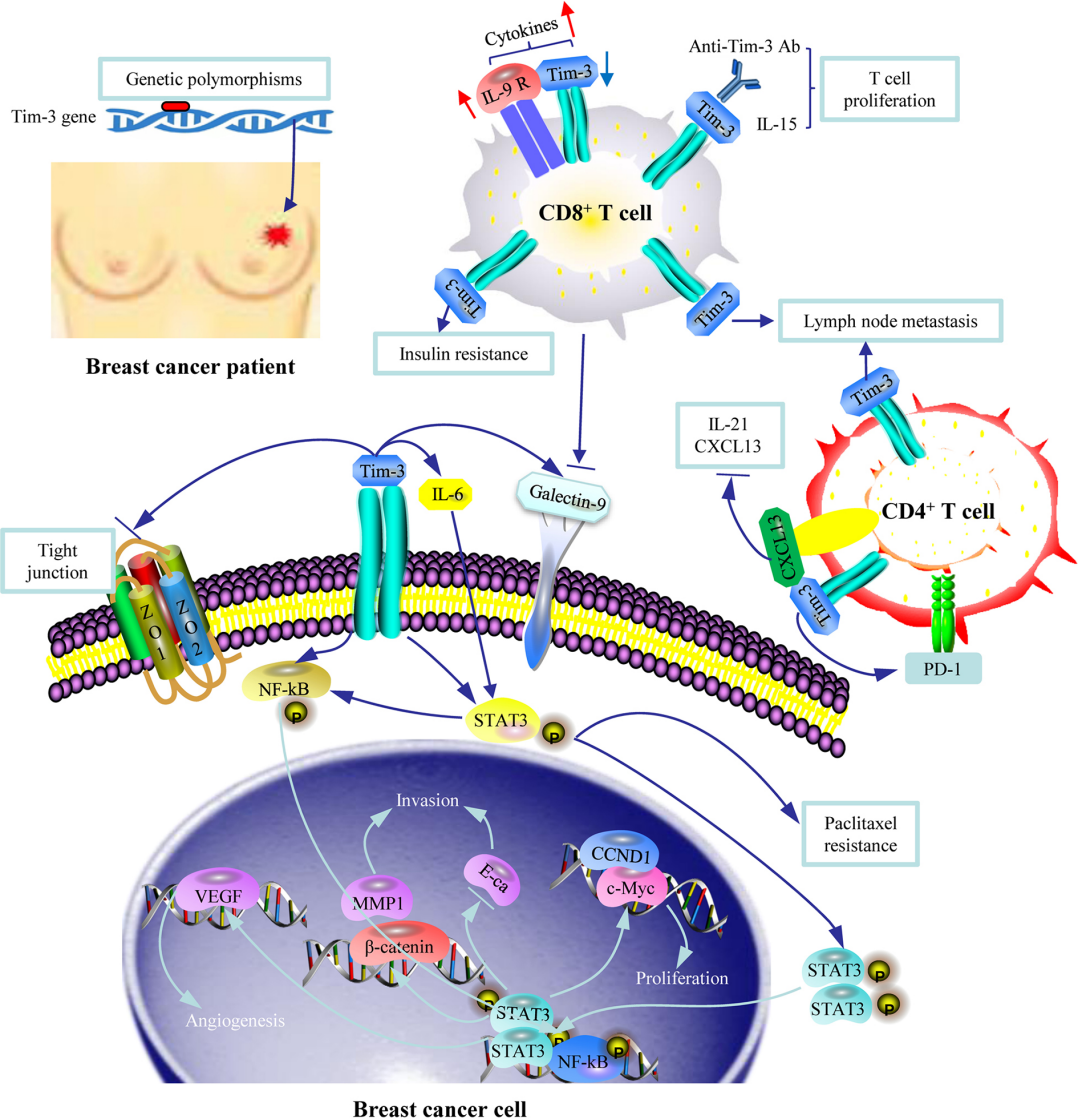
An illustration of the role of Tim-3 in breast cancer. Genetic polymorphisms in Tim-3 are associated with susceptibility to breast cancer. Tim-3^+^ CD8^+^ T cells and Tim-3^+^ CD4^+^ T cells are associated with risk of lymph node metastasis through Tim-3 mediated immune escape. Tim-3 is involved in insulin resistance. Tim-3 overexpression in breast cancer cells promotes cell proliferation, migration, invasion, tubal formation, and enhanced chemoresistance to paclitaxel by activating the NF-κB/STAT3 signaling pathway. The Tim-3-galectin-9 (Gal-9) pathway is involved in tumor progression given the surface-based Gal-9 protects breast carcinoma cells against cytotoxic T cell-induced death.

Tim-3 gene polymorphism is also involved in breast cancer susceptibility and disease progression. The rs10053538 GT+TT genetic variant of Tim-3 is associated with increased genetic predisposition to breast cancer and faster progression (26). The rs10053538 GT+TT genotype is associated with higher Tim-3 expression and increased lymph nodes metastasis (26). Another study showed that the +4259T/G SNP in the Tim-3 gene is a genetic risk factor for the progression and prognosis of invasive breast cancer (27). This study reported a significantly higher prevalence of the +4259T/G genotype and the +4259G allele among patients with breast cancer than among the controls. Moreover, the +4259T/G polymorphism correlated with a higher expression of the cell proliferation index, Ki-67, in patients with metastasis than those without (27). Therefore, genetic polymorphisms in Tim-3 also play a critical role in breast cancer tumorigenesis and progression (**Figure 1**), which is likely because Tim-3 could suppress the immune response of T cells to tumors.

## The Role of Tim-3 Expression on Immune Cells in Breast Cancer

Tim-3 was initially considered to be expressed only by T cells. However, it is now known to be expressed by multiple cell types including T cells (21, 28), dendritic cells (DCs) (29), macrophages (30), myeloid-derived suppressor cells (MDSCs) (31), NK cells (10), stromal cells (19), and vascular endothelial cells (32). Comparing the results of single-cell RNA sequence analysis between breast cancer and normal cells demonstrates that Tim-3 is predominantly expressed on myeloid cells (33). The expression of Tim-3 on multiple immune cell types explains its widespread inhibition in the tumor microenvironment.

A study including 109 patients with TNBC reported expression of Tim-3 in tumor-infiltrating lymphocytes (TILs) from all patients including 17 with <5% stained TILs, 31 with 6%-25% stained TILs, 48 with 26%-50% stained TILs, and 13 with >51% stained TILs (34). In this study, a higher Tim-3 level significantly correlated with younger patients, high proportion of TILs, higher tumor stage, high PD-1 and PD-L1, but with a positive prognosis (34). Two studies further assessed the role of Tim-3 expressed on intra-epithelial TILs (iTILs) and stromal TILs (sTILs) in breast cancer. One study showed that patients with breast cancer with Tim-3^+^ iTILs (≥ 1%) represent a minority of cases (11%), with a predilection for basal-like breast cancers. Tim-3^+^ sTILs (≥2%) represented 20% of cases and included more non-basal cases. The presence of Tim-3^+^ iTILs was highly correlated with stromal TILs and other immune checkpoint markers (PD-1^+^ iTILs, LAG-3^+^ iTILs and PD-L1^+^ tumors) (35). Another study showed that luminal A and luminal B breast cancer were associated with higher expression of Tim-3 in sTILs compared to HER2-positive and triple-negative subtypes, but without effect on disease-free survival (DFS) (36) (**Supplementary Table 1**).

Several studies have explored the effect of Tim-3 on CD8^+^ T cells for it plays a central role in mediating anti-tumor immunity. Tim-3 expression on CD8^+^ T cells was higher in invasive ductal carcinoma tissue than in normal tissue and correlated with lymph node metastasis, WHO grade, and molecular subtypes in cancer (21). Another study assessed the association of Tim-3 expression on T cells from tumor-draining lymph nodes with breast cancer progression. The authors reported that the frequency of Tim-3^+^ CD8^+^ T cells was associated with a higher tumor grade and was significantly higher in patients with more involved lymph nodes than in those with fewer involved nodes (37). The underlying mechanism for this could be the Tim-3-mediated inhibition of the proliferation and activation of CD8^+^ T cells. Another study supported this view, reporting that IL-15-costimulated tumor infiltrating CD8^+^ T cells exhibited stronger early proliferation and IFN-g production, which attenuated in the later stages owing to the upregulation of Tim-3 signaling (38). Addition of the Tim-3 ligand Gal-9 significantly suppressed IL-15 costimulation, whereas blocking Tim-3 enhanced it (38). Moreover, compared to IL-9R low CD8^+^ T cell subset, the IL-9R high subset was characterized by a lower expression of inhibitory molecules Tim-3, PD-1, and killer cell lectin-like receptor G1 (KLRG-1) *ex vivo* and lower IFN-γ after stimulation, which may render the IL-9R high CD8^+^ T cells less susceptible to signaling mediated by inhibitory ligands, thus leading to higher cytokine expression (39). In addition, Tim-3 on naïve and central memory (CM) CD8^+^ T subsets is associated with breast cancer insulin resistance (IR). IR+ patients presented a significantly lower PD-1^+^ Tim-3^-^ frequency in CD8^+^ T subsets compared to those without (40) (**Figure 1**) (**Supplementary Table 1**).

The expression of Tim-3 in CD4^+^ T cells was also upregulated in breast cancer (28), and correlated with metastatic lymph node load (37), suggesting its importance in suppressing the immune microenvironment. The Tim-3 level in circulating Tfh (CD4^+^CXCL13^+^follicular helper T) cells in patients with breast cancer was significantly elevated, which was a Tfh exhaustion marker. Compared to Tim-3^−^ Tfh cells, Tim-3^+^ Tfh cells expressed a higher level of PD-1, decreased chemokine CXCL13 and cytokine IL-21 levels, and contained fewer proliferating cells. Naive B cells cocultured with Tim-3^+^ Tfh cells resulted in significantly lower IgM, IgG, and IgA expression than those cocultured with Tim-3^-^ Tfh cells, demonstrating that a reduction in Tim-3^+^ Tfh required B cell involvement. Moreover, the percentage of Tim-3^+^ Tfh cells in resected breast tumor tissues was much higher than in autologous blood, which also suggests a participation of Tim-3^+^ Tfh cells in tumor microenvironment (41) (**Figure 1**).

Another study revealed that Tim-3 expression was also localized to macrophages and cDCs in tumors and normal tissues, with the highest levels consistently found on the CD103^+^ cDC1 subset (29). Tim-3 expression by intratumoral CD103^+^ DCs regulates chemokine expression during paclitaxel treatment and promotes paclitaxel resistance (29) (**Supplementary Table 1**).

## The Role of Tim-3 Expression on Tumor Cells in Breast Cancer

Tim-3 is not only expressed on immune cells but also overexpressed in multiple types of malignant tumors, such as lung cancer (42), gastric cancer (43), colon cancer (44), hepatocellular carcinoma (45), renal cell carcinoma (46), bladder urothelial carcinoma (47), cervical cancer (48), and breast cancer (20, 21, 49). The ubiquitous expression of Tim-3 on tumor cells strongly indicates its potential role in tumor progression. A meta-analysis showed that a high expression of Tim-3 in solid tumors is associated with a significantly shorter OS (50). However, a high level of Tim-3 expression is associated with better prognosis in several tumor types. Tim-3 expression in renal cell carcinoma is associated with longer progression-free survival and OS (51), whereas low Tim-3 expression levels in tumor tissue is associated with poor prognosis in metastatic prostate cancer (52). Similarly, downregulation of Tim-3 promotes invasion and metastasis of colorectal cancer cells (53). These seemingly contradictory findings imply tumor-type dependent role of Tim-3, which necessitates exploring the role of Tim-3 in breast cancer.

Studies have shown that the expression of Tim-3 on breast cancer cells was significantly higher compared to that on normal tissue [98% *vs* 13% (21), and 42.9% *vs* 18.2% (20)]. Tim-3 expression level on tumor cells was correlated with age ≥45 years (21), greater number of axillary lymph node metastases (21), more advanced clinical stage (20, 21), higher Ki-67 index (20), and a lower 5-year survival (20). Based on the different molecular biology of breast cancer, it would be desirable to explore the expression of Tim-3 in tumors by subtype as well as in primary and metastatic tumors in the future. Several basic research studies explored the mechanism underlying the negative role of Tim-3 in breast cancer. Tim-3 overexpression in breast cancer cells promotes cell proliferation, migration, invasion, and tumor-associated tubal formation and enhances chemoresistance to paclitaxel by activating the NF-κB/STAT3 pathway and its downstream genes (cyclin D1, matrix metalloproteinase-1, vascular endothelial growth factor, and E-cadherin). Tim-3 also deteriorates tight junctions by downregulating zona occludens (ZO)-2, ZO-1, and occludin, which further accelerates tumor progression (54). Another study supported the aforementioned findings by reporting that downregulation of Tim-3 in breast cancer cells inhibited their proliferation, migration, and invasion and promoted their apoptosis (20). Furthermore, breast tumors expressed higher levels of both Tim-3 and Gal-9 compared to healthy tissues, and these proteins were colocalized. The surface-based Gal-9 could protect breast cancer cells against cytotoxic T cell-induced cell death (49) (**Figure 1**).

The expression of Tim-3 in tumors could also interact with immune cells in tumor microenvironment (TME) and promote tumor progression. STAT3 signaling was shown to pay a role in immune cells and promoted immunosuppressive function in the TME (55). Tim-3 overexpression in breast cancer cells activated the STAT3 signal pathway, and then maybe converged in both tumor promotion and immunosuppression, such as the crosstalk between tumor cells and immune cells (56).

## The Role of Tim-3 in Breast Cancer Prognosis

The immune microenvironment is strongly correlated with the prognosis of cancer, even in the early-stage ductal carcinoma *in situ* (57). A meta-analysis including 7284 patients with different types of malignant tumors suggested that Tim-3 is an independent prognostic factor for poor OS (58). However, the prognostic role of Tim-3 in breast cancer is different depending on the type of Tim-3 expressing cells.

A study on the effect of the gene expression level of Tim-3 on breast cancer survival by analyzing the KM-plotter database revealed that patients with high Tim-3 expression had a significantly worse relapse-free survival (RFS). OS displayed a similar trend but without statistical significance (20). Another study described a 7 nuclear receptors-based risk score which could effectively predict breast cancer OS. In this study, immune cell infiltration differed significantly between the high-risk and low-risk groups, of which Tim-3 and PD-1 were enhanced in the high-risk group, indicating that the poor prognosis of patients in the high-risk group could be because of the suppression of the immune microenvironment (59). However, another study analyzed the RNA-seq data in the Cancer Genome Atlas (TCGA) database and found that overexpression of Tim-3 correlated with improved OS in breast cancer (17). The differing prognostic outcomes could be attributed to the differing associations between Tim-3 expression with prognostic outcomes by breast cancer subtype. This hypothesis is supported by a subgroup analysis which showed that a high Tim-3 level was associated with worse RFS in luminal A and luminal B subtypes, but improved RFS in basal breast cancer. With regard to OS, high Tim-3 levels were associated with a worse prognosis in luminal A subtype but a better prognosis in the basal subtype of breast cancer (20). Supporting this observation, another study showed that elevated Tim-3 expression significantly correlated with improved RFS in estrogen receptor (ER)-negative or progesterone receptor (PR)-negative breast cancer (17). Meanwhile, other studies showed that both gene (19, 60) and protein (33) expression levels of Tim-3 had no association with survival in patients with breast cancer. These results may partly explain the poor efficacy of anti-immunization checkpoint drugs used as monotherapies in the treatment of breast cancer.

The expression of Tim-3 on immune cells also affects the prognosis in breast cancer. Although a higher expression of Tim-3 on TILs is associated with poor clinical and pathologic features, such as younger patients, high tumor stage, high PD-1, and high PD-L1, patients with high Tim-3 in TILs have better DFS and OS in TNBC (34). Similarly, another study reported the presence of Tim-3^+^ iTILs as an independent favorable prognostic factor in the whole cohort and among ER-negative patients (35). In contrast, Tim-3 positivity in stromal regions after neoadjuvant chemotherapy (NAC) was significantly associated with poor prognosis in TNBC (61).

Tim-3 also has predictive value for the therapeutic outcomes in breast cancer. Patients with a high level of Tim-3 expression had more favorable survival outcome after adjuvant chemotherapy or systemic treatments than those with a low level of expression. Of note, increased Tim-3 expression was significantly associated with better RFS in patients treated with chemotherapy than those not (17). This could be partly attributed to the expression of Tim-3 being significantly associated with infiltrating immune cells such as infiltrating CD8^+^ T cells, T cells (general), B cells, monocytes, and tumor-associated macrophages (TAMs) (17).

## Regulation of Tim-3 Expression in Breast Cancer

Understanding the regulatory mechanisms of Tim-3 in breast cancer would be of great value for future research and treatment strategy. Previously reported molecules or transcription factors affecting Tim-3 expression include T-bet (62), MEK (63), c-Jun (64), and nuclear factor interleukin 3 regulated (65) in T cells, T-bet in HCV-infected monocytes or macrophages (66), Hif1-α in brain damage (67), and CB2 cannabinoid receptors in ischemic microglial cells (68). Recently, few novel regulating mechanisms of Tim-3 in breast cancer have been identified.

Several novel factors including micro-RNA, cytokines, TNF receptors, and chemotherapy were recognized to regulate Tim-3 expression in immune cells. Treatment of CD8^+^ T cells with a miR-149-3p mimic attenuated markers of T-cell exhaustion and downregulated mRNAs encoding Tim-3, PD-1, B- and T-lymphocyte attenuator and Forkhead Box P1 (69). In contrast, T-cell proliferation and activation cytokines (IL-2, TNF-α, and IFN-γ) were upregulated after treatment with the miR-149-3p mimic. Treatment with an miR-149-3p mimic reverses CD8^+^ T cell exhaustion and promotes the CD8^+^ T lytic activity on 4T1 mouse breast tumor (69). Tumor-secreted cytokines also regulate the expression of Tim-3 in T cells. Tim-3 expression significantly increased on activation of prostaglandin E2 (PGE2) and cyclic AMP signaling pathways. A study revealed elevated Tim-3 expression in Jurkat T cells on exposure to breast tumor cell-conditioned media through the interaction between PGE2 and its receptor EP4 (70). Another study revealed that glucocorticoid-induced TNF receptor expressed in lymphocytes in breast cancer was associated with immune checkpoint markers (Tim-3, PD-1, PD-L1and LAG-3) and T-cell markers (CD8 and FoxP3), indicating that it could also regulate the expression of Tim-3 (71). Ly6GmiLy6Clo CD11b^+^ CXCR2^+^ subpopulation (CXCR2^+^ MDSCs) predominantly proliferates and is recruited in the tumor microenvironment during breast cancer progression. CXCR2^+^ MDSCs promote breast cancer progression by directly inducing cancer cell epithelial-mesenchymal transition and indirectly promoting T-cell exhaustion by upregulating the expression of immunosuppressive molecules Tim-3, PD-1, PD-L1, LAG-3, and CTLA-4 on CD4^+^ or CD8^+^ T cells and inducing exhaustion of the activated T cells *via* IFN-γ (72). A study reported that plasma concentrations of some immune checkpoint markers varied as a function of age: Tim-3, Gal-9, and sCD25 levels were elevated, whereas 4-1BB (CD137) and PD-L1 levels were attenuated in advanced age (73). Furthermore, Victor Sarradin et al. evaluated the immune biomarkers of paired pre- and post-NAC tumor samples in the tumor (no-pathologic complete response, no-pCR) or tumor bed area (pCR), and found that Tim-3 positivity (≥ 1%) was significantly increased after NAC with increases occurring more frequently in no-pCR than in pCR TNBC patients (51.4% *vs* 31%) (61). Another study showed fewer CD4^+^ T-cells expressing Tim-3 and increased PD-1 and Tim-3 expression on CD8^+^ T cells following NAC (74). This observation could be attributed to the differences in the activation status of circulating CD4^+^ and CD8^+^ T cells after NAC or differences in the effect of chemotherapeutic drugs on cytokine production by the T cells (74).

**Figure 2:**
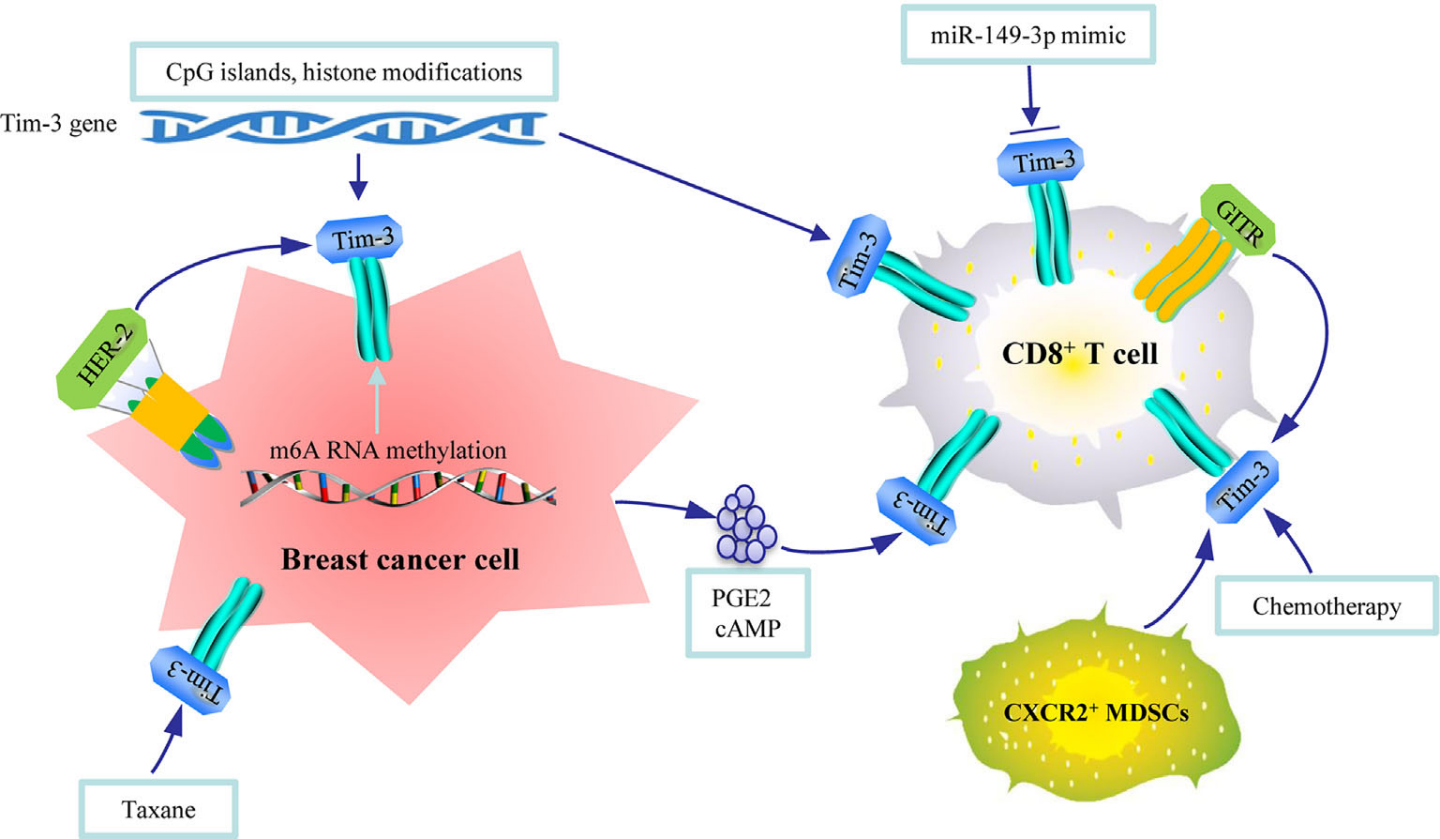
An illustration of the regulation mechanisms of Tim-3 in breast cancer. CpG islands and histone modifications in Tim-3 gene, N6-methyladenosine (m6A) RNA methylation, epidermal growth factor receptor 2 epidermal growth factor receptor 2 (HER2) expression, and taxane can regulate the expression of Tim-3 in breast tumor tissues. Micro RNA, cytokines released by tumor cells, TNF receptor, MDSCs, and chemotherapy can regulate the expression of Tim-3 in immune cells.

Several studies have also evaluated the regulatory factors of Tim-3 in breast cancer tissues, including CpG islands, N6-methyladenosine (m6A) RNA methylation, and chemotherapy. Vertebrate CpG islands represent a dispersed but coherent DNA sequence class whose members function as genomic platforms for regulating transcription at their associated promoters (13). CpG islands in the promoter region of Tim-3 were significantly hypomethylated in breast tumor tissue than in normal tissue (18). In addition, decreased binding of H3K9me3 and H3K27me3 was observed in the promoter loci of Tim-3 in tumor tissues. Therefore, both DNA and histone modifications are involved in the upregulation of Tim-3 in breast tumor tissue (18). However, in peripheral blood mononuclear cells of patients with breast cancer, PD-L1 and TIGIT expressions could be regulated by DNA methylation epigenetic machinery; however, no changes in Tim-3, CTLA-4, and LAG-3 expressions were observed compared to those in healthy donors (75). N6-methyladenosine (m6A) RNA methylation plays critical roles in tumorigenesis and cancer immunoregulation (76). By analyzing the RNA sequencing data of 24 main m6A RNA methylation regulators in patients with breast cancer from TCGA, 2 subgroups of RNA methylation (RM1 and RM2) were identified. Of the 2, RM2 presented greater RNA methylation modification compared to RM1, and RM2 was associated with significantly better OS. One of the reasons why RM2 was associated with better prognosis was because RM2 was associated with higher expressions of HLA-A and higher numbers of tumor-infiltrating CD8^+^ T cells, helper T cells, and activated NK cells but lower expressions of Tim-3, PD-L1, PD-L2, and CC chemokine receptor 4 (CCR4). The aforementioned results suggest that m6A RNA methylation could regulate the expression of Tim-3 in breast cancer (77). Chemotherapy could also regulate the expression of Tim-3 in breast cancer. Using whole-transcriptome sequencing and whole-exome sequencing with 37 metastatic breast cancer samples, the authors found that HER2 expression and taxane treatment correlated positively with a high expression of HAVCR2 (Tim-3), PDCD1 (PD-1), CD274 (PD-L1), CD276 (B7-H3), CTLA-4, indoleamine 2,3-dioxygenase 1(IDO1), and LAG-3 (78), supporting that HER2 expression and taxane treatment could regulate the expression of Tim-3 in breast cancer (**Figure 2**).

## The Therapeutic Significance of Tim-3 in Breast Cancer

In recent years, immune checkpoint blockade and vaccines administered in combination with other treatments have emerged as potential breast cancer treatments (79). ICIs, either alone or in combination with other therapies, have created new paradigm in tumor treatment (79). ICIs have significant advantage over conventional therapies, but only a fraction of patients benefit from the current ICIs, and the response rates remain relatively low (80). The coblockade of PD-1 and PD-L1 upregulates the coexpression of Tim-3 and LAG-3 on CD4^+^ CD25^+^ T cells and CD4^+^ CD25^+^ FoxP3^+^ Helios^+^ Tregs in TNBC, indicating that the emergence of compensatory inhibitory mechanisms leads to acquired TNBC resistance against PD-1/PD-L1 blockade (28). Therefore, current research efforts are exploring the possible beneficial effects of blocking Tim-3 as a therapy for cancer.

A few basic research studies suggest that blocking Tim-3 may have remarkable therapeutic value in breast cancer. Tim-3 expression is significantly upregulated on γδ T cells during their *ex vivo* expansion, and these γδ T cells with overexpressed Tim-3 exhibit an increased susceptibility to apoptosis. The combined use of a Tim-3 inhibitor and MT110 (anti-CD3× anti-EpCAM) could enhance the anti-tumor effect of the adoptively transfused γδ T cells, which have clinical implications for the design of new anti-tumor regimens (81). Another study revealed that the outgrowing transgenic T cells exhibit an exhausted phenotype characterized by PD-1 and Tim-3 upregulation and failed to control tumor growth in the absence of costimulatory signals. However, by coexpressing 2G CAR.MUC1 (signal 1-activation + signal 2-costimulation) and 4/7ICR (signal 3-cytokine), transgenic T cells selectively expanded at the tumor site and produced potent and durable tumor control (82). In addition, inhibiting or blocking Tim-3 enhances the effect of chemotherapy for breast cancer. The study by de Mingo Pulido et al. (29) showed that intratumoral CD103^+^ DCs highly express Tim-3. Anti-Tim-3 antibody promotes CXCL9 expression by these DCs, which enhances the function of CD8^+^ T cells and thereby improves paclitaxel’s therapeutic effect in breast cancer murine models of triple-negative and luminal B diseases. Another study reported similar findings that the combination of paclitaxel and *Ganoderma lucidum* spores exhibited improved tumor control through recovery of the exhausted TILs by inhibiting the expressions of immune checkpoints (Tim-3 and PD-1), whereas paclitaxel alone evidently increased CTLA-4 expression (83).

**Figure 3:**
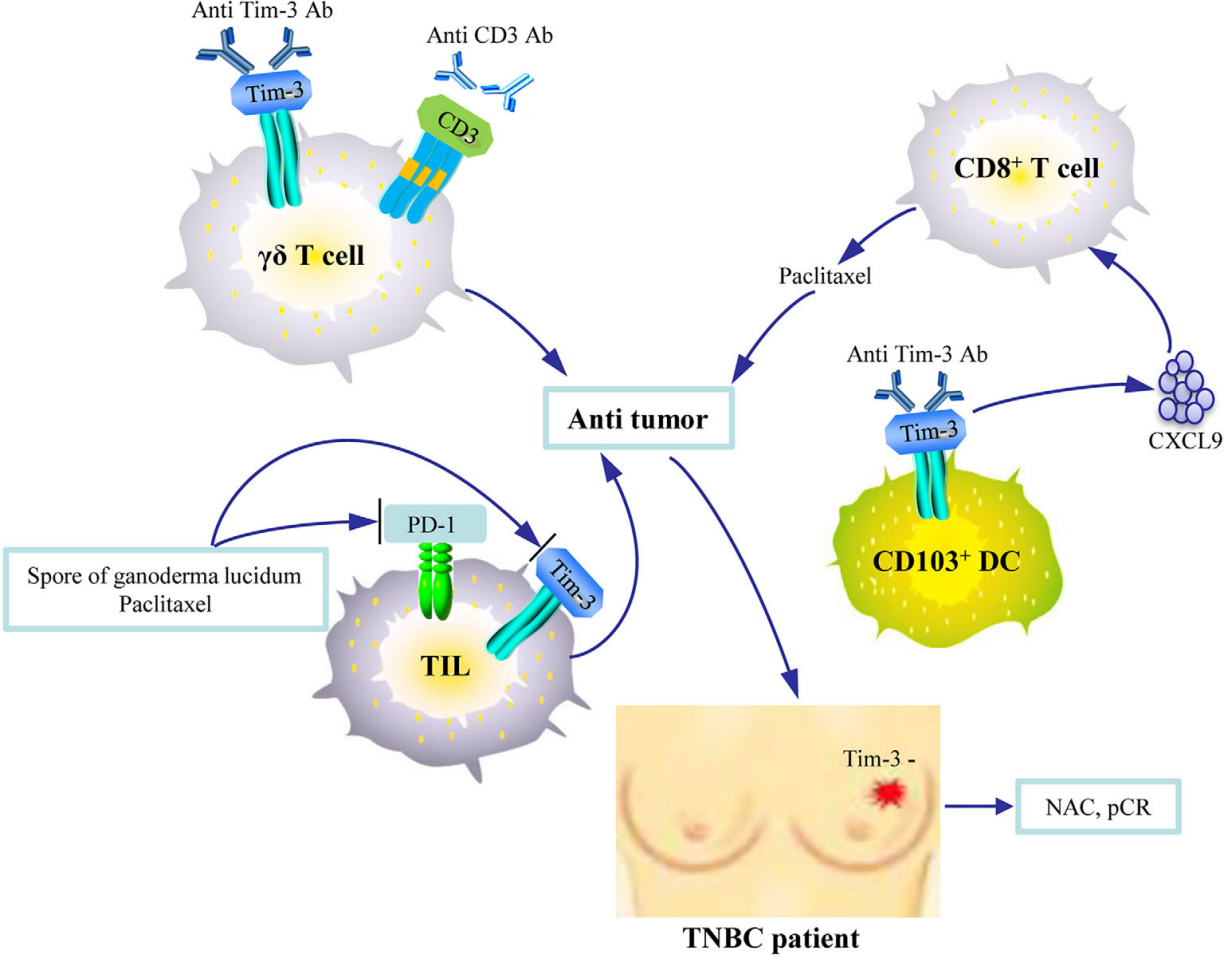
An illustration of the therapeutic significance of Tim-3 blockade in breast cancer. The combined use of Tim-3 inhibitors and anti-CD3 agents enhances the anti-tumor activity of the adoptively transfused γδ T cells. Anti-Tim-3 antibody promotes CXCL9 expression by CD103^+^ DCs, which enhances the function of CD8^+^ T cells and improves paclitaxel’s therapeutic activity. The combination of paclitaxel and *Ganoderma lucidum* spores enhances tumor control by allowing the recovery of the exhausted tumor infiltrating lymphocytes (TILs) by inhibiting Tim-3. Tim-3-negativity on TILs is associated with pathologic complete response (pCR) after neoadjuvant chemotherapy (NAC) in patients with triple-negative breast cancer (TNBC).

Another study reported that, clinically, patients with increased plasma Tim-3 or CTLA-4 expression after treatment initiation experience greater benefit from camrelizumab (anti-PD-1 immune checkpoint inhibitor) with apatinib (vascular endothelial growth factor receptor-2 inhibitor) in advanced TNBC (84). Furthermore, Tim-3-negativity is significantly associated with a pCR after NAC, whereas Tim-3 positivity on TILs is associated with a worse chemotherapy response (85) (**Figure 3**). These findings indicate that combined immune checkpoint inhibitor therapies *via* Tim-3 blockage may increase sensitivity to chemotherapy and enhance its effect (8, 11). However, Tim-3^+^ CD8^+^ was not associated with pCR in paired breast cancer samples before and after NAC in a prospective cohort (n = 50) (86). Therefore, further study is required to evaluate the therapeutic effect of blocking Tim-3 in breast cancer. Several ongoing prospective and planned clinical trials have been initiated in solid tumors with several Tim-3 antibodies, including TSR-022 (NCT02817633), MBG453 (NCT02608268), and LY3321367 (NCT03099109) (10, 87). Most of these anti-Tim-3 antibodies are being tested in combination with anti-PD-1/PD-L1 mAbs. Bispecific antibody targeted at both Tim-3 and PD-1 is also being tested (88). Importantly, early data have shown that the combination is well tolerated without dose-limiting toxicity. The results of these ongoing clinical trials should be significant contributions to breast cancer therapy in the future.

So far, most studies indicated that blocking Tim-3 may have remarkable anti-tumor effect. A recent study performed an RNA sequencing analysis and explored the changes in signaling pathway caused by Tim-3 blockade in tumor-infiltrating immune cells (80). The results show that Tim-3 blockade enhances anti-tumor immunity by upregulating genes through means such as acetylation, cell differentiation, apoptosis, TGF-β signaling, immune response, negative regulation of angiogenesis, activation of the IFN-γ-mediated pathway, and mitogen-activated protein kinase signaling that favor immune cell proliferation and activation and enhance T-cell cytotoxicity (80). Furthermore, it suppresses tumor angiogenesis, growth, invasion, and metastasis by downregulating genes associated with transcriptional regulation, integrins, cell proliferation, cancer related-pathways, JAK-STAT signaling, angiogenesis, negative regulation of apoptosis, and Wnt signaling (80). These novel findings further our understanding of the pathways regulated by Tim-3 in breast cancer and provide valuable insights for future research.

## Conclusion and Perspective

Tim-3 is broadly expressed by different types of cells in breast cancer. It has critical roles in tumorigenesis, tumor progression and predicting prognosis. The biology of Tim-3 is complex depending on the cells it is expressed on or the molecular subtypes of breast cancer. Several novel factors including micro-RNA, cytokines, TNF receptors, CpG islands, N6-methyladenosine (m6A) RNA methylation, and chemotherapy are identified to regulate the expression of Tim-3. Tim‐3 blockade induces anti‐tumor immune response, inhibits tumor growth, and enhances the effect of chemotherapy. Therefore, Tim-3 in breast cancer could be a promising target in tumor treatment.

Currently, the therapeutic potential of targeting Tim-3 is being studied in solid tumors. Tim-3 coblockade with other checkpoint receptors is being investigated in clinical studies, and promising results have been reported in patients with anti-PD-1-refractory disease. Therefore, activating cell costimulatory molecules by combining anti‐Tim-3 antibodies with other ICIs or with chemotherapy may be of great potential in improving the treatment outcomes of breast cancer in the future. In addition, recent studies showed that simultaneously block TGF-β and PD-L1 pathways had a superior anti-tumor effect compared to the monotherapies (89, 90). YM101, a bispecific antibody that bound to TGF-β and PD-L1, could effectively counteract the biological effects of TGF-β and PD-1/PD-L1 pathway and enhance the anti-tumor activity *in vivo* (89). Similarly, using M7824 to simultaneously target TGF-β and PD-L1/PD-1 immunosuppressive pathways promoted anti-tumor responses and efficacy in murine breast and colon carcinoma models (90). Therefore, based on the above encouraging findings, it may also have potential for developing the anti-TGF-β/Tim-3 bispecific antibody to conquer the resistance to immune checkpoint inhibitors for cancer patients in the future.

## Author Contributions

YC and GQ conceived and planned the article. YC and JL took the lead in writing the manuscript in consultation with GQ. GC contributed to the drawing the figures. All authors contributed to the article and approved the submitted version.

## Funding

The study was supported by Shandong Medical and Health Science and Technology Development Project (No. 202004081034), Special fund for clinical research of Wu Jieping Medical Foundation (No. 320.6750.2020-20-4), and Yantai Science and Technology Innovation Development Plan Project (No. 2021YD007).

## Conflict of Interest

The authors declare that the research was conducted in the absence of any commercial or financial relationships that could be construed as a potential conflict of interest.

## Publisher’s Note

All claims expressed in this article are solely those of the authors and do not necessarily represent those of their affiliated organizations, or those of the publisher, the editors and the reviewers. Any product that may be evaluated in this article, or claim that may be made by its manufacturer, is not guaranteed or endorsed by the publisher.
